# Expression of Interferon Effector Gene SART1 Correlates with Interferon Treatment Response against Hepatitis B Infection

**DOI:** 10.1155/2016/3894816

**Published:** 2016-12-18

**Authors:** Yong Li, Chuanlong Zhu, Faxi Wang, Tiantian Zhu, Jun Li, Shufeng Liu, Fei Xiao

**Affiliations:** ^1^Department and Institute of Internal Medicine, Tongji Hospital, Tongji Medical College, Huazhong University of Science and Technology, Wuhan 430030, China; ^2^Department of Infectious Diseases, The First Affiliated Hospital of Nanjing Medical University, Nanjing 210029, China; ^3^The Center for Biomedical Research Center, Tongji Hospital, Tongji Medical College, Huazhong University of Science and Technology, Wuhan 430030, China; ^4^Center for Immunology and Infectious Diseases, Biosciences Division, SRI International, Harrisonburg, VA 22802, USA; ^5^Department of Medicine, Division of Infectious Diseases and Geographic Medicine, Stanford University School of Medicine, Stanford, CA 94305, USA; ^6^Department of Microbiology and Immunology, Stanford University School of Medicine, Stanford, CA 94305, USA

## Abstract

Interferon-*α* (IFN-*α*) has limited response rate in the treatment of chronic hepatitis B (CHB). The underlying mechanism of differential responsiveness to IFN remains elusive. It has been recently reported that SART1 mediates antiviral effects of IFN-*α* in the hepatitis C virus (HCV) cell culture model. In this study, we investigated the role of SART1 in antiviral activity of IFN-*α* against hepatitis B virus (HBV) using blood and liver biopsy samples from chronic hepatitis B patients treated with pegylated IFN-*α* and HepG2 cells transfected with cloned HBV DNA. We observed that the basal SART1 expression in liver and PBMCs before IFN treatment was significantly higher in responders than in nonresponders. Furthermore, baseline SART1 expression level positively correlated with the degree of HBV DNA and HBeAg decline after IFN treatment. Mechanistically, silencing SART1 abrogated the antiviral activity of IFN-*α*, reduced the expression of IFN-stimulated genes (ISGs) Mx, OAS, and PKR, and attenuated JAK-STAT signaling in HepG2 cells, suggesting that SART1 regulates IFN-mediated antiviral activity through JAK-STAT signaling and ISG expression. Our study elucidates the important role of SART1 in IFN-mediated anti-HBV response and provides new insights into understanding variation of IFN treatment response in CHB patients.

## 1. Introduction

Hepatitis B remains a global health problem with 350–400 million people being chronically infected with hepatitis B virus worldwide [1, 2]. These patients are prone to life threatening complications such as HBV acute-on-chronic liver failure (ACLF) [3], cirrhosis, and hepatocellular carcinoma (HCC) [4]. There are currently two classes of agents approved for the treatment of chronic hepatitis B (CHB): nucleos(t)ide analogues and standard or pegylated interferon-*α* (Peg-IFN-*α*) [5]. IFN-*α* is an important cytokine of the innate immune and adapt immune response embracing both immunomodulatory and antiviral activity [4–6]. IFN-*α* binds to its receptor, activates the JAK-STAT signaling pathway, and transcriptionally induces interferon-stimulated genes (ISGs) including “classical ISGs” myxious resistance protein (MxA) [7], 2,5-oligoadencylate sythase (OAS), and RNA-dependent protein kinase (PKR) [8, 9], which have been found to mount antiviral effect against HBV and other viruses [10]. Although substantial progress has been made in treatment of hepatitis B in the past decade, less than 30% of the CHB patients show sustained response to IFN therapy.

Although IL-28B genotype, HBV genotype, serum ALT, and HBV DNA levels prior to treatment have been found to affect the response of IFN treatment [11–14], the relationship between host gene and IFN response of HBV treatment remains unclear. Squamous cell carcinoma antigen recognized by T cells (SART1) was reported as a U4/U6 · U5 tri-snRNP specific factor with specific E3 ubiquitin ligase activity, playing a key role in recruiting tri-snRNP in the spliceosome assembly [15]. It regulates cell proliferation and therefore has potential to be used as a target in cancer therapy [16–18]. Recent studies found that SART1 exerted its antiviral activity through enhancing ISG expression using HCV cell culture models [19, 20]. Since ISG induction also restricts HBV infection [21, 22], we hypothesize that SART1 plays a role in regulating ISG expression against HBV infection after IFN treatment.

In this study, we identified a previously unrecognized function of SART1 during HBV treatment. We report for the first time that SART1 expression correlates with IFN-*α* treatment response in CHB patients. Furthermore, silencing SART1 abrogated the antiviral effect of IFN-*α* on HBV replication and decreased expression of the IFN-*α* downstream ISGs in HBV cell models. Our study reveals the role of SART1 in IFN-*α* treatment of CHB and provides potential biomarker to predict IFN treatment outcome in HBV infection.

## 2. Materials and Methods

### 2.1. Study Subjects, Clinical Samples, and Study Design

The study was conducted in accordance with the guidelines of the Declaration of Helsinki and received prior approval from the Institutional Review Boards of The First Affiliated Hospital of Nanjing Medical University, Nanjing, China. Patient's written informed consent was obtained prior to inclusion in the study. Thirty-three naïve CHB patients were recruited from 2012 through 2014 at The First Affiliated Hospital of Nanjing Medical University. Inclusion criteria for naïve CHB patients in the study were HBeAg positivity and HBV DNA level > 10^4^ copies/mL. All the patients receiving treatment were injected with Peg-IFN-*α* 180 *μ*g weekly for 48 weeks. Blood samples and PBMCs of all IFN-*α* treated patients were collected at baseline and 12 weeks following IFN-*α* treatment. Liver biopsy samples were collected 24 h prior to IFN treatment. We defined virological response as HBV DNA level < 1000 copies/mL, serological response as HBeAg loss or seroconversion, biochemical response as normalization of ALT level, and a combined response as HBV DNA level < 1000 copies/mL and HBeAg or HBsAg loss. Remaining patients who could not meet the criteria were classified as nonresponders. We assessed both treatment endpoint combined responses after 48 weeks of Peg-IFN-*α* therapy and sustained combined responses at 24 weeks after the treatment endpoint. Characteristics of the patients are presented in Table 1.

### 2.2. Markers Characterizing HBV Infection

HBV DNA was measured using a real-time PCR (Amplicor HBV Monitor Test, Roche Diagnostics, Mannheim, Germany). HBsAg and HBeAg levels were measured with the ARCHITECT HBsAg assay (Abbott Laboratories, Lake Forest, IL, USA) and AxSYM HBe 2.0 assay (Abbott), respectively. Anti-HBs and anti-HBe were determined by ARCHITECT qualitative assays (Abbott). Serum biochemical markers and alanine aminotransferase (ALT) were assayed using an Automatic Serum Analyzer (HITACHI 747, Japan).

### 2.3. Plasmids and Reagents

pHBV-1.3 was generated from the HBV genome as described [22]. Interferon stimulated response element luciferase reporter plasmid (pISRE-luc) was kindly provided by Professor Lin wenyu (Massachusetts General Hospital, Harvard Medical School, Boston). Specific small interfering RNA (siRNA) against SART1 (siSART1), IFNAR (siIFNAR), and siRNA control were purchased from RIBOBIO Biotech (Guangzhou, China).

### 2.4. Cell Cultures

The human hepatoma cell line HepG2 was obtained from American Type Culture Collection. HepG2 cells were grown in Dulbecco's modified Eagle's medium (DMEM) supplemented with 10% fetal bovine serum (Gibco BRL, Gaithersburg, MD) at 37°C with 5% CO_2_. Cells were plated in 12-well or 6-well plate depending on the experiment and were grown to 60%–70% confluence prior to transfection.

### 2.5. Luciferase Reporter Assay and Transfection

HepG2 cells were reverse-transfected with the indicated siRNA in 12-well plates for 24 h before plasmid transfection using Lipofectamine 2000 (Invitrogen). Interferon stimulated response element (ISRE) mediated IFN signaling was monitored by dual-luciferase reporter assay system after cotransfecting the pISRE-luc plasmids expressing firefly luciferase and pRL-TK plasmids expressing Renilla luciferase as an internal control. Forty-eight hours after p-ISRE transfection, 1000 IU/mL IFN-*α* was added and incubated with the cells for 8 hours as described by others [23–26]. Relative luciferase activity was assessed by the Promega dual-luciferase reporter assay system (Pro-Omega, Madison, WI). Relative luciferase unit (RLU) was calculated by dividing the firefly luciferase value by the Renilla luciferase value.

### 2.6. Quantification of HBV e-Ag and HBV Surface Ag

Cells were transfected as indicated and were cultured for an additional 24 h in DMEM without FBS or antibiotics. The conditioned media were collected, and a standard ELISA kit was used to quantify HBV e-Ag (HBeAg) and HBV surface Ag (HBsAg) (Shanghai KeHua Biotech, Shanghai, China).

### 2.7. qRT-PCR

Cell culture RNA and RNA from PBMCs of CHB patients were extracted with Trizol reagent (Invitrogen, Carlsbad, CA) and reverse-transcribed into cDNA with KR-103 reverse transcriptase (Tiangen, Beijing, China). Expression levels were quantified by qRT-PCR performed on a 7500 HT qRT-PCR system (Applied Biosystems, Life Technologies, Darmstadt, Germany) using the CT method. Relative mRNA levels of all target genes were normalized to house-keeping gene (GAPDH) levels. The sequences of primers for target genes are shown in Table 2.

### 2.8. Western Blot Analysis

Proteins of target gene were prepared as previously described [27]. The protein concentration of each sample was determined with BCA Protein Assay Kit P0012S (Beyotime, China). Proteins were separated by stacking gel and SDS-PAGE with a Tris glycine system at 100 V for 90 min and transferred to polyvinylidene fluoride membranes (Millipore, USA). The membranes were blocked in 3% nonfat dry milk in phosphate-buffered with nonfat dry milk. After being incubated with primary antibody for 1-2 hours, the membranes were washed three times and incubated with horseradish peroxidase-conjugated secondary antibody. Blots were developed with ECL western blotting substrate (Thermo Pierce, Rockford, IL). Antibodies (Abs) against MxA and *β*-actin were purchased from Santa Cruz Biotechnology (Santa Cruz, CA), Abs against SART1, STAT1, p-STAT1, STAT2, and p-STAT2 were purchased from SAB Technology (Signalway-Antibody, USA), and Abs against PKR and OAS were purchased from PTG LAB (Rosemont, IL). Band intensities were quantified by gel-pro software.

### 2.9. Immunohistochemistry

Liver sections (>4 mm) were cut from paraffin blocks and then treated with 3% H_2_O_2_, permeabilized with 0.5% triton, and incubated with 3% bovine serum albumin (BSA). The samples were stained with rabbit anti-human SART1 polyclonal antibody (1 : 100) at 37°C for 45 minutes, followed by incubation with the HRP-conjugated goat anti-rabbit mAb (Boshide, Wuhan, China) at 37°C for 30 minutes according to the instruction of the immunohistochemistry kit (SP9001; Zhongshan Biotechnology, Beijing, China).

### 2.10. Statistical Analysis

Statistical analysis was performed by the Student's *t* test or Mann-Whitney *U*-test, as appropriate. The expression of target gene and its correlation with clinical markers was processed by Pearson correlation. All statistical analyses were carried out with SPSS v.11 (SPSS, Chicago, USA). *p* < 0.05 was considered statistically significant.

## 3. Results

### 3.1. Pretreatment SART1 Expression Levels Correlate with IFN-*α* Response in Patients with CHB

**Figure 1:**
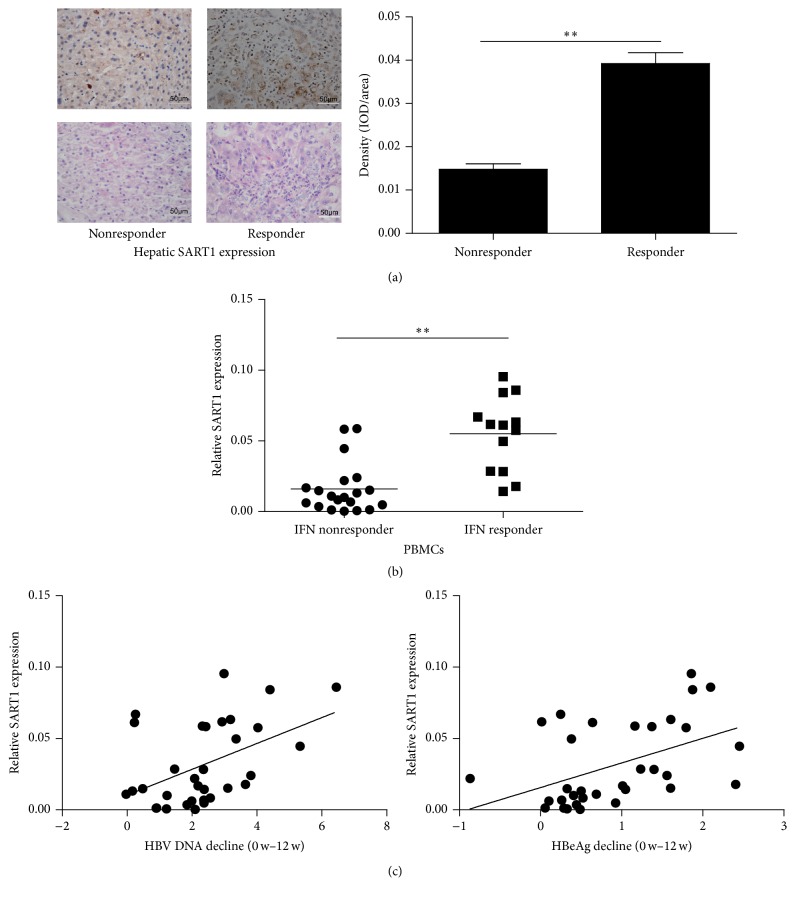
Expression of SART1 gene correlates with interferon treatment outcome. (a-b) SART1 expression in both liver (a) and blood (b) between IFN responder and nonresponders prior to Peg-IFN-*α* treatment was evaluated by immunohistochemical staining of liver (a) and qPCR (b). The intensity of positive staining in tissue sections was analyzed by integrated optical density (IOD) shown as histograms and is represented as means ± SD from three experiments performed in triplicate. Student's *t*-test (a) and Mann-Whitney *U*-test (b) were used to determine the significance of the differences between these two groups, respectively, ^*∗∗*^ *p* < 0.01. (c) Correlation of SART1 baseline expression with HBV DNA (left panel; *r* = 0.47, *p* < 0.05) or HBeAg decline (right panel; *r* = 0.49, *p* < 0.05) from 0 to 12 weeks was evaluated by Pearson correlation coefficient.

Using immune-histochemical staining and qRT-PCR, we first analyzed the expression of SART1 in both live biopsy and PBMCs from CHB patients before IFN-*α* treatment. Interestingly, hepatic SART1 baseline level expression in responders was significantly higher than in nonresponders (Figure 1(a)). The SART1 mRNA expression in PBMCs of IFN responders was also significantly higher than that of nonresponders (Figure 1(b)). Furthermore, among all the CHB patients receiving IFN treatment, pretreatment SART1 expression in PBMCs was positively associated with HBV DNA and HBeAg decline from 0 to 12 weeks (Figure 1(c)). These results demonstrate that SART1 expression correlates with virological response of IFN treatment in CHB patients, indicating that SART1 plays a key role in antiviral effect of IFN against HBV.

### 3.2. Silencing SART1 Restricts the Antiviral Activity of IFN-*α*

**Figure 2:**
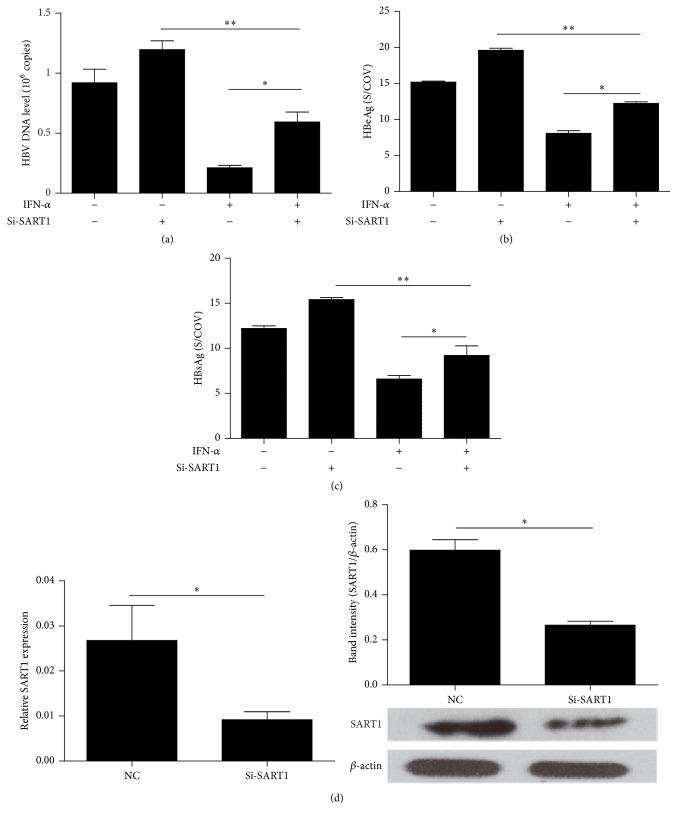
Silencing SART1 antagonizes the antiviral activity of IFN-*α*. (a–c) HepG2 cells were cotransfected with HBV-1.3 and Si-SART1 for 24 h. The cells were then treated with 1000 IU/mL IFN-*α* for 24 h. 48 h after transfection, the supernatants were collected and assayed for quantification of HBV DNA by real-time PCR, HBeAg, and HBsAg by ELISA. An siRNA-negative control was used as a control. (d) HepG2 cells were transfected with si-SART1. 48 h after transfection, the efficiency of si-SART1 was detected by qRT-PCR and western blot. Means ± SD from three independent experiments performed in triplicate are shown. Student's *t*-test was used to determine significance of differences between two groups. ^*∗*^ *p* < 0.05, and ^*∗∗*^ *p* < 0.01.

To further investigate the role of SART1 in IFN-mediated antiviral activity, we knocked down SART1 in HBV replication cell culture system using siRNA. Specific siRNA targeting SART1 (si-SART1) was transfected along with pHBV-1.3 into HepG2 cells (50–60% transfection efficiency), followed by treatment of 1000 IU/mL exogenous IFN-*α* 24 h posttransfection. Forty-eight hours after transfection, HBV infection was evaluated by HBV DNA replication as well as HBeAg and HBsAg secretion. As shown in Figures 2(a)–2(c), HBV DNA replication, HBeAg, and HBsAg secretion were detected in HBV replication cell culture model. IFN-*α* significantly inhibited HBV DNA replication as well as HBeAg and HBsAg secretion. Silencing SART1 not only increased HBV infection but also compromised antiviral activity of IFN, indicating that SART1 exerts IFN-associated antiviral activity in HBV replication cell model. The efficiency of SART1-specific siRNA was shown by measuring SART1 mRNA and protein in Figure 2(d). Notably, IFN still imposes significant antiviral effect after SART1 knockdown, indicating that there are additional factors/pathways other than SART1 regulating IFN's antiviral effect.

### 3.3. Silencing SART1 Suppresses the Expression of IFN-*α* Downstream Antiviral Effectors

**Figure 3:**
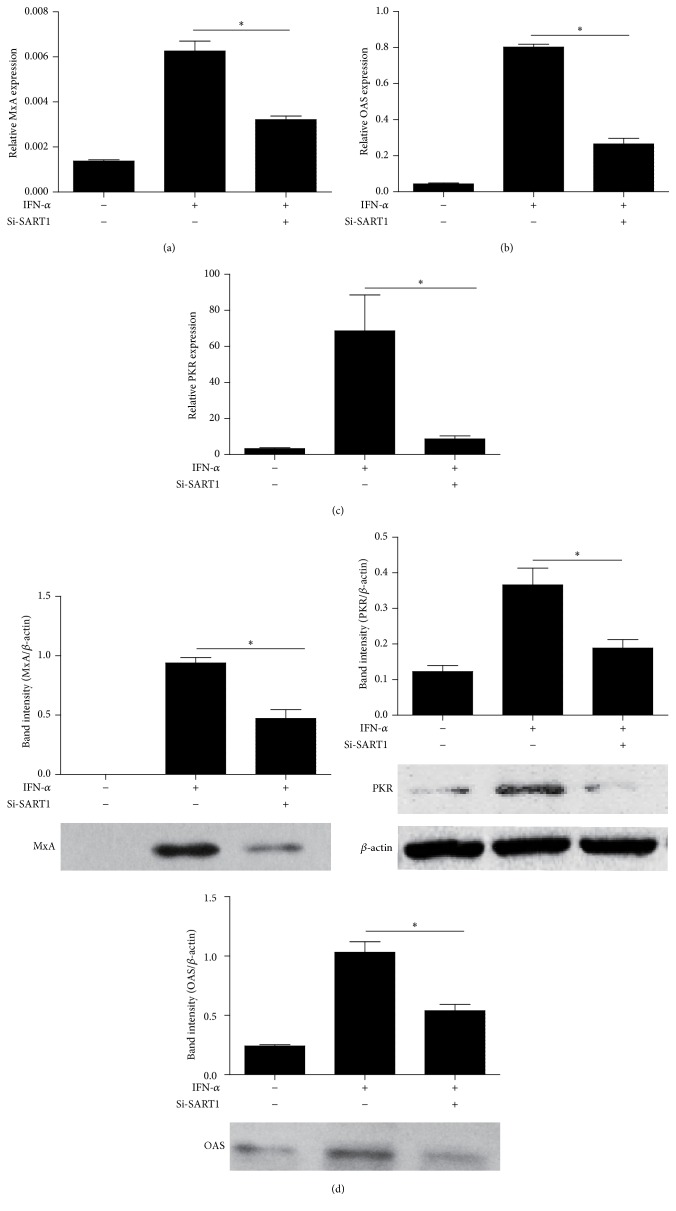
Silencing SART1 decreases the expression of IFN-*α* downstream effectors. (a, b, c) HepG2 cells were transfected with si-SART1 or si-NC for 24 h and then treated with 1000 IU/mL IFN-*α* for 24 h. 48 h after transfection, the mRNA levels of MxA, OAS, and PKR were analyzed by qRT-PCR. ^*∗*^ *p* < 0.05. (d) HepG2 cells were transfected with Si-SART1 or empty vector for 24 h and then treated with 1000 IU/mL IFN-*α* for 24 h. 48 h after transfection, the protein levels of MxA, OAS, and PKR were analyzed by western blotting. Means ± SD from three independent experiments performed in triplicate are shown. Student's *t*-test was used to determine significance of differences between two groups. ^*∗*^ *p* < 0.05.

Mx, OAS, and PKR are classical downstream ISGs with antiviral activity. These ISG-encoded proteins interfere with distinct steps in viral replication or trigger the degradation of viral RNA and protein to exert antiviral activity. To explore the role of SART1 in modulating these downstream IFN effectors, we transfected HepG2 cells with si-SART1 before IFN-*α* treatment. IFN-induced MxA, OAS, and PKR expression levels were measured by qRT-PCR and western blot. As shown in Figure 3, SART1 knockdown reduced both mRNA and protein levels of IFN-induced MxA, OAS, and PKR. These results demonstrate that silencing SART1 abrogates antiviral activity of IFN through downregulating ISGs expression.

### 3.4. Silencing SART1 Abrogates IFN Anti-HBV Efficacy by Attenuating JAK-STAT Signaling

**Figure 4:**
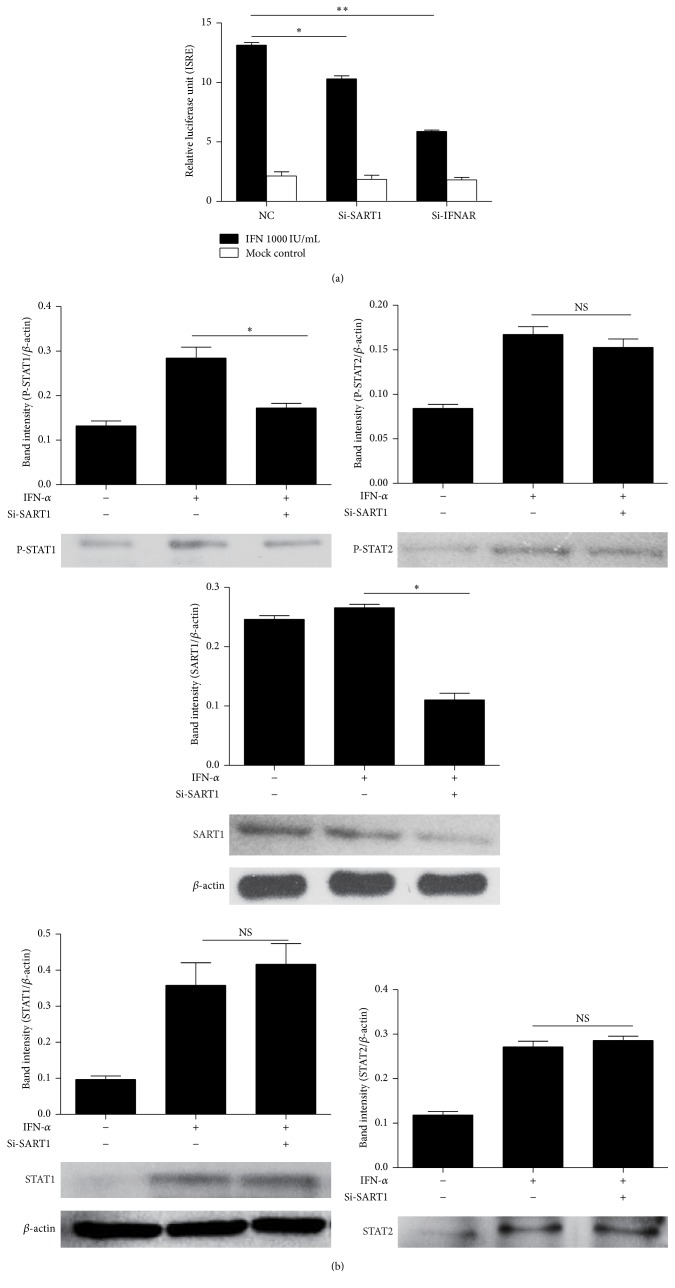
Effect of SART1 on ISRE activity and IFN-induced JAK-STAT signaling. (a) HepG2 cells were reverse-transfected with the indicated siRNA 24 h before p-ISRE and pRL-TK cotransfection. 1000 IU/mL IFN-*α* was added 48 h later. Relative luminescence activity (RLA) was measured 8 h later. (b) HepG2 cells were transfected with Si-SART1 for 48 h and treated with 1000 U/mL IFN-*α* for 24 h (for STATs) or 1 h (for p-STATs) before cells were harvested. The expression of STAT1, STAT2, p-STAT1, and p-STAT2 was determined by western blotting. Means ± SD from three independent experiments performed in triplicate are shown. Student's *t*-test was used to determine significance of differences between two groups. NS = nonsignificant, ^*∗*^ *p* < 0.05, and ^*∗∗*^ *p* < 0.01.

To further investigate the mechanistic action of SART1 in regulating ISGs expression, we assessed the interaction among SART1, ISGs, and JAK-STAT signaling using a luciferase reporter system driven by the IFN-stimulated response element (ISRE). ISRE-driven luciferase activity increased more than three times upon IFN-*α* stimulation in the presence of nontargeting siRNA (siCTRL NC). Knockdown of SART1 by si-SART1 significantly reduced ISRE activity compared with control cells (Figure 4(a)), implying that SART1 inhibits ISGs expression through downregulating ISRE activity. To gain insights into the effect of SART1 on IFN-induced JAK-STAT signaling, we next investigated STAT phosphorylation after SART1 knockdown. As shown in Figure 4, knockdown of SART1 significantly reduced STAT1 phosphorylation and slightly decreased STAT2 phosphorylation (Figure 4(b)). By contrast, silencing SART1 had no effect on the IFN-induced expression of total STAT1 and STAT2. Taken together, these results indicate silencing SART1 attenuates IFN-induced JAK-STAT signaling followed by downregulation of ISGs expression.

## 4. Discussion

Although IFN-*α* and its pegylated form (Peg-IFN-*α*) have been used as a first line therapy of chronic hepatitis B for more than 20 years, the sustained response rate to IFN treatment remains far from satisfying [26–28]. Data from clinical trials showed that 32% HBeAg-positive patients achieved HBeAg seroconversion and 14% patients obtained undetectable viral load after IFN treatment [29]. Therefore, multiple efforts have been exerted on predicting response to IFN therapy to individualize treatment. The underlying mechanism of responsiveness to IFN remains unknown, resulting in difficulties improving prediction of IFN treatment outcome and making individualized treatment recommendations. We and others recently reported that SART1 transcriptionally regulates the antiviral activity of IFN-*α* against HCV using an HCV cell culture model [19, 20]. However, the function of SART1 was investigated only in the HCV cell culture model. To further explore the role of SART1 in IFN-*α*'s antiviral effect, in this study we used liver and PBMCs samples from CHB patients and the HBV replication cell culture model in the hope of elucidating the mechanism of responsiveness to IFN and identify potential biomarkers to predict IFN treatment outcome in HBV infection.

We first checked basal expression of SART1 in both liver biopsy samples and peripheral PBMCs from CHB patients prior to IFN-*α* treatment. Interestingly, the SART1 expression in IFN responders was significantly higher than in IFN nonresponders. Thus, higher SART1 baseline levels strongly suggest a better response to IFN-*α* during HBV treatment. Importantly, SART1 baseline expression of PBMCs displayed a trend for positive correlation with HBV DNA and HBeAg decline from 0 to 12 weeks, respectively. These data suggest that SART1 is an IFN-associated antiviral host gene against HBV. In this scenario, it supports the hypothesis that SART1 plays an important role and might be used as a biomarker in the response to IFN-*α* treatment.

To investigate the mechanism of SART1 involving in IFN-*α*'s antiviral activity, it is important to correlate SART1 expression and IFN-*α* anti-HBV effect. We knocked down SART1 in HBV replication cell culture model and found that silencing SART1 abrogated IFN-*α*'s suppressive effects against HBV replication in HepG2 cells transfected with HBV1.3 plasmid. Combined with our* in vivo* study, the association of SART1 expression and the IFN-*α* anti-HBV effect in the HBV cell model and CHB patients was persuasive. Next, we make an effort to explore the mechanism by which SART1 regulated the IFN-mediated anti-HBV effect, which is dependent on key ISGs expression including antiviral protein MxA, OAS, and PKR [30]. MxA proteins were initially discovered in influenza A resistant mice and are thought to mediate innate immunity against numerous viruses. Its anti-HBV mechanism has been well characterized in MxA overexpressing mice [31]. OAS proteins are able to synthesize oligoadenylates that activate the latent form of RNaseL and then trigger the degradation of viral RNA [32]. PKR belongs to protein kinases that act in an IFN-*α* and dsRNA-dependent mechanism through inhibition of translation by the phosphorylation of EIF2a [9]. We then asked whether SART1 mediated IFN-*α*'s anti-HBV effects through regulating ISGs mentioned above. Interestingly, we detected the decreased ISGs mRNA and proteins expression in HepG2 cells treated with IFN-*α* upon silencing SART1. These results are consistent with the previous reports on HCV [19]. Therefore, our study further reinforces the findings of earlier studies, indicating that SART1 regulated IFN-*α* antiviral activity not only in HCV infection but also in HBV infection. Moreover, our* in vitro* study indicates that SART1 mediates IFN antiviral effects against HBV through regulation of downstream ISGs expression.

It is worth pointing out that there are multiple factors regulating IFN's antiviral effect as previously reported, such as apolipoprotein B mRNA editing enzyme catalytic polypeptide-like 3 (APOBEC3), epidermal growth factor receptor, and nuclear factor *κ*B [26, 33, 34]. This is supported by our data, which show that knockdown of SART1 only partially blunts IFN's antiviral effect.

To further study the detailed mechanism of the SART1-mediated IFN anti-HBV effect, we focused on the IFN signaling pathway. The binding of IFN-*α* to type I IFN receptor activates the JAK-STAT signaling pathway, resulting in dimerization of STAT1 and STAT2 in association of IFN regulatory factor 9 to form IFN-stimulated gene factor 3 complex, which translocates to the nucleus and binds to ISRE to promote transcription of ISGs [35, 36]. Thus, we sought to determine intersection of SART1 with the IFN signaling pathway by using a luciferase reporter system driven by the ISRE. As expected, SART1 had effects on ISRE activity compared to nontargeting siRNA. Importantly, silencing SART1 had no effect on the IFN-induced expression of total STAT1 and STAT2 but significantly reduced STAT1 phosphorylation and slightly decreased STAT2 phosphorylation. Thus, we show that SART1 indeed plays an important role in IFN-*α* signaling.

Admittedly, there are some limitations in our study. First, the number of patient samples is relative small, which could be solved by expanding study subjects in future studies. Second, further studies are needed to explore the mechanism of different SART1 expression in CHB patients. IL28B single nucleotide polymorphisms, rs12979860 T/C and rs8099917 T/G, have been reported to associate with spontaneous and treatment-induced viral clearance in HCV infection [37–39]. SART1 promoter polymorphism and its effect on SART1 transcription, binding of transcription factors to the polymorphism region, and so forth are worth investigating [40].

In conclusion, our study provides new insights into the SART1-mediated regulation of ISGs and response in CHB patients with IFN treatment. The variation of IFN-*α*'s antiviral response is associated with differential SART1 gene expression level in PBMCs and liver. However, the biological mechanisms underlying the influence of SART1 on response of IFN-*α* treatment need to be further investigated. Investigation of the mechanism of SART1-mediated regulation on ISGs and IFN-*α*'s antiviral effect, both* in vivo* and* in vitro*, may provide a better understanding of the role of SART1 in HBV innate immune response to viral infection and novel strategies that might be employed to develop antiviral treatment.

## 5. Conclusions

In summary, we discovered that SART1 expression correlates with IFN treatment responses in CHB patients and the HBV replication cell culture model. Mechanistically, SART1 regulates IFN-mediated antiviral activity through JAK-STAT signaling and ISG induction. Our findings may be beneficial for providing a potential biomarker of IFN treatment response in CHB disease management.

## Figures and Tables

**Table 1:** Baseline characteristics of patients included in the study.

Characteristics	Responders	Nonresponders
Age, y	27 ± 6.62	27.77 ± 3.44
Gender, M/F	10/3	17/3
HBV DNA (Log^10^ copies/mL)	8.03 ± 1.03	8.38 ± 1.37
HBeAg (Log^10^ PEIU/mL)	2.24 ± 1.31	2.4 ± 1.15
HBsAg (Log^10^ IU/mL)	3.71 ± 0.91	4.17 ± 0.78
ALT (IU/mL)	140.55 ± 92.48	178.23 ± 153.35

**Table 2:** Sequences of qRT-PCR primers for target genes.

Genes	Forward	Reverse
GAPDH	cggatttggtcgtattggg	ctcgctcctggaagatgg
SART1	gaaccttgtggcttctcttca	gtcatccactgcccattagg
MxA	acctgatggcctatcaccag	tgaagaactggatgatcaaagg
OAS	gacggatgttagcctgctg	tggggatttggtttggtg
PKR	aaagcgaacaaggagtaag	gatgatgccatcccgtag

## Abbreviations

ALT: Alanine aminotransferase
CHB: Chronic hepatitis B
HBeAg: HBV e-Ag
HBsAg: HBV surface Ag
HBV: Hepatitis B virus
MxA: Myxovirus resistance protein
OAS: 2,5-Oligoadenylate synthetase
PKR: Protein kinase R
qRT-PCR: Quantitative RT-PCR
SART1: Squamous cell carcinoma antigen recognized by T cells
si-IFNAR: siRNA against interferon-*α* receptor
si-SART1: siRNA against SART1
siRNA: Small interfering RNA.
